# Nutrition education strategies to promote vegetable consumption in preschool children: the Veggies4myHeart project

**DOI:** 10.1017/S1368980021004456

**Published:** 2022-04

**Authors:** Cátia Braga-Pontes, Sara Simões-Dias, Marlene Lages, Maria P Guarino, Pedro Graça

**Affiliations:** 1 ciTechCare – Centre for Innovative Care and Health Technology, Polytechnic Institute of Leiria, Leiria, Portugal; 2 ESSLei – School of Health Sciences, Polytechnic Institute of Leiria, 2411-901 Leiria, Portugal; 3 EpiDoC Unit, CEDOC, NOVA Medical School, Universidade Nova de Lisboa, Lisboa, Portugal; 4 Faculty of Nutrition and Food Sciences of University of Porto, Porto, Portugal

**Keywords:** Child, Preschool, Vegetables, Health education, Video games

## Abstract

**Objective:**

To test the efficacy of three nutrition education strategies on the intake of different vegetables in preschool children.

**Design:**

This is an experimental study conducted in four Portuguese preschools. The intervention consisted of 20-min educational sessions, once a week, for 5 weeks, with one of the following randomised educational strategies: Portuguese Food Wheel Guide (control), digital game, storybook, storybook and reward (stickers). All groups had repeated exposure to vegetables in all sessions. A pre- and post-test were conducted to determine vegetable intake, and a 6-month follow-up was realised.

**Setting:**

Preschools of Leiria district, Portugal.

**Participants:**

A sample of 162 children aged 3 to 6 years. All eligible children attending the preschools were invited to participate.

**Results:**

All interventions tested were effective in increasing vegetable consumption both in the short and medium term, without statistically significant differences, compared to the control group. Stickers were more effective in the short term than in the medium term.

**Conclusions:**

The nutritional education strategies associated with repeated exposure tested in this study were effective in promoting vegetable consumption in preschool children. The use of stickers may be a valid strategy to promote the consumption of vegetables less recognised by children.

Good nutrition during the first years of life is crucial for optimising growth and development of children as well as to ensure good health in the short and long term. Child’s nutrition is particularly important, not only for its role in preventing chronic diseases but also because many of the eating habits that start in childhood will persist into adulthood^(1)^. Fruits and vegetables have a prominent role in dietary recommendations due to their high concentration of vitamins, minerals, phytochemicals, particularly antioxidants, and also because they are a great source of dietary fibre^(2)^. Although it is known that the consumption of vegetables has a more protective effect on health than fruit, it remains a great challenge to increase the consumption of vegetables, especially among children^(3,4)^. In Portugal, the data presented by the National Food Survey in 2017 shows that 52·7 % of the Portuguese population do not consume more than 400g/d of fruit and vegetables. In children, this percentage of non-compliance increases to 68·9 %, with a particular lower consumption of vegetables compared to fruit^(5)^.

Most European countries have implemented campaigns to promote the consumption of fruit and vegetables in various population groups^(6)^. The WHO considers the promotion of healthy food consumption in schools one of the priority axes for reducing obesity before the age of 5 years, reinforcing the importance of well-structured and effective food and nutrition education programmes^(7)^. School-based food education projects should be designed to create a healthy eating preference learning environment, either through repeated and sustained exposure to healthy foods, consistent and comprehensive meal standardisation, and food education activities aimed at promoting the literacy and skills of children, teachers and food service workers^(8,9)^. With preschool children it is fundamental to use playful activities to promote learning, since the act of playing is a natural act, being the child’s motivation a facilitator of learning^(10)^. In this context, gamification strategies and digital games (DG) have been increasingly used to promote healthy behaviours and have proved to be valid strategies^(11,12)^. According to the gamification concept, a properly designed virtual game can encourage the player to consume vegetables by earning virtual rewards or reaching a more advanced level of play^(13)^. Gamification applied to nutrition can be defined as the strategy of employing game design elements (points, badges, leaderboards, progress bars, performance graphs or avatars) in interventions to improve eating behaviour^(14)^. A systematic review of games for health identified 1743 games produced between 1983 and 2016, of which 5·47 % were about nutrition education and eating disorders^(15)^. Although there are still few publications aimed at evaluating the game elements that promote the effectiveness in nutritional behaviours, evidence suggests that games may influence dietary intake and have generally positive effects on nutrition knowledge^(11)^. In this review, three studies have found that exposure to 5 min of play influences the child eating behaviour immediately after playing, which suggests the possible behavioural impact of dietary game play^(11)^. Another strategy used in preschool age is story reading. The stories, through their narrative or illustrations, can transmit information and emotions that may be related to the feeding process, from the origin of the food to its contribution to a healthy life^(16)^. The involvement that is established with books, sensory exploration and the interaction resulting from reading a story are fundamental components for a high-quality educational programme at an early age^(17)^. Previous studies with preschool children have shown that the use of picture books with food has increased the willingness of children to taste the foods represented in the story^(3)^. Another food education strategy used with preschool children is repeated exposure. This strategy has been extensively studied and shown to be consistent to increase food consumption, especially in the case of novel or disliked vegetables, via familiarisation and learned safety^(18)^. Generally, five to ten exposures are necessary to increase intake^(3)^. Further strategies like modelling, flavour enhancement, stealth, tangible rewards (e.g. stickers) or social praise have been shown to promote vegetable intake^(19)^. According to a systematic review and meta-analysis of strategies to increase vegetable consumption in preschool children, tangible rewards coupled with taste exposure are slightly more effective than taste exposure coupled with social rewards^(20)^. Additionally, the Food Dudes programme, a randomised controlled trial and multi-component intervention to promote fruit and vegetable consumption in children, revealed that tangible rewards were more effective than social rewards both in the short and long term^(21)^.

Combining nutrition education carried out with instruments that are specifically designed to increase knowledge about eating vegetables (DG and storybook (SB)) with taste exposures might produce a concomitant effect in increasing vegetable intake^(3,11,18,22)^. The opportunity for preschool children to explore the different vegetables (taste, smell and texture) combined with learning about their characteristics and health benefits can boost consumption and preference for this type of food^(23)^. In Portugal, there are few guidelines for food education at preschool age and there are limited studies evaluating the impact of food education at these ages. Therefore, this study aims to test the efficacy of three nutrition education strategies – DG, SB, storybook and use of stickers as a reward (SBS) – on the intake of five different vegetables in preschool children, compared to the control group – Portuguese Food Wheel Guide (PFWG).

## Methods

The Veggies4myHeart project is an experimental study conducted in four Portuguese preschools to test the efficacy of three educational strategies: (1) DG, (2) SB and (3) SBS, compared to the gold standard of nutrition education in Portugal, the PFWG. The PFWG was revised and launched in 2003 and the Ministry of Health freely distributed Food Wheel posters and related materials through all public schools and preschools. Thus, all the preschool children in Portugal have access to the Food Wheel.

A pre- and post-test were conducted to determine the differences between groups in vegetable intake. A 6-month follow-up was conducted to determine whether any of the effects found were maintained.

**Fig. 1:**
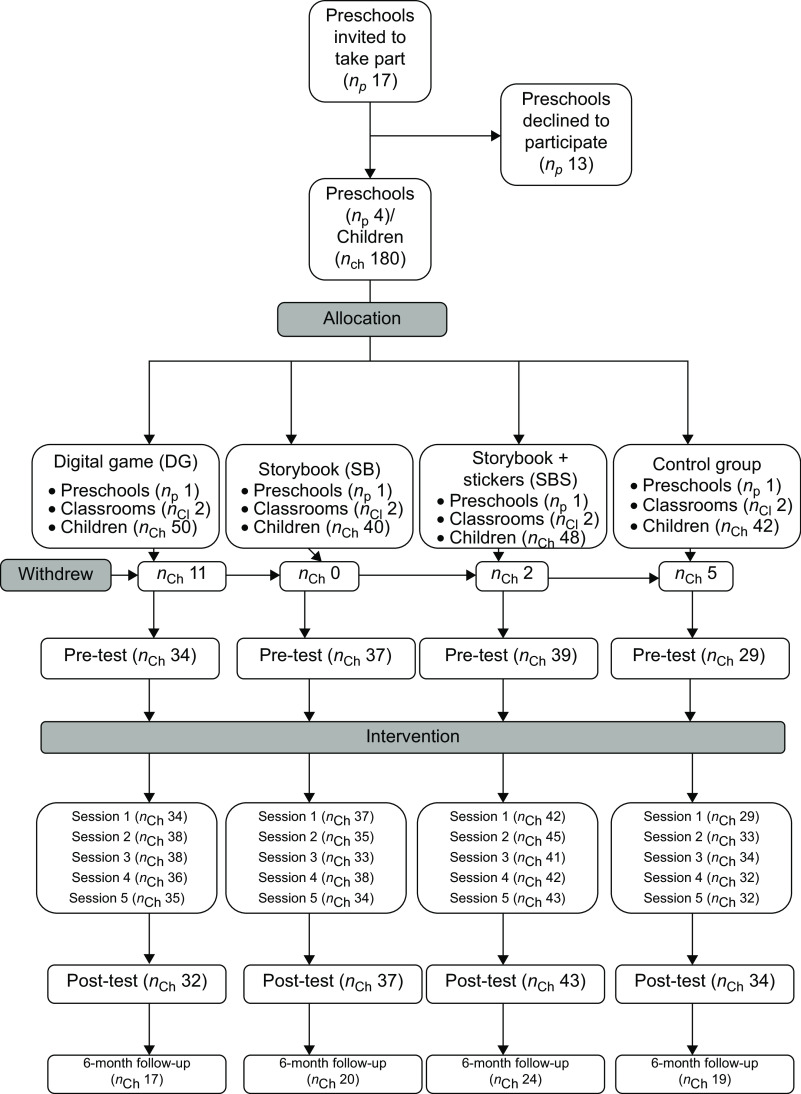
Flowchart methods

The study design is set out in Fig. 1.

### Recruitment

Seventeen public preschools in the district of Leiria (Portugal) were invited to take part in the study. Four preschools with a total of eight classrooms accepted to participate and were randomly assigned to one of the four groups of the study by the research team. Due to the serendipitous matching of participant schools with the number of interventions, randomisation was carried out by draw, whereby the four interventions were drawn to the four schools that agreed to participate.

### Participants

Participants were all children aged 3 to 6 years who attended the public preschools selected for the study and who provided informed consent by their caregivers.

Caregivers from all groups completed a written questionnaire about socio-demographic conditions before the beginning of the intervention. The household elements description was obtained by questioning the guardian caregiver on ‘how many people live in the same house as the child?’. Children included in the assessments were those who attended preschools on the date when the assessment sessions were conducted and data were collected from April to June 2019. They were excluded from the study if they did not want to participate at the time of assessments. The vegetable tasting did not replace any pre-existing meal or snack time. Instead, vegetable tasting moments occurred during class time, in moments chosen by the teachers as the most appropriate one to cause the less disturbance possible. Table 1 shows the intervention design and the respective timeline.

**Table 1:** Design and timeline of the Veggies4myHeart project

Condition	Baseline (baseline)	Intervention + assessment of five vegetable consumption (lettuce, tomato, carrot, purple cabbage and cucumber)					Post-test	Follow-up
		Target vegetable						
Digital game (DG)	Assessment of vegetables knowledge + assessment of five vegetables consumption	Tomato	Purple cabbage	Cucumber	Carrot	Lettuce	Assessment of vegetables knowledge + assessment of five vegetables consumption	Assessment of vegetables knowledge + assessment of five vegetables consumption
Storybook (SB)								
Storybook and stickers (SBS)								
Control group								
Timeline	Week 1	Week 2	Week 3	Week 4	Week 5	Week 6	Week 7	Week 29

### Intervention

The intervention consisted of 20-min educational sessions once a week for 5 weeks. Educational sessions were realised in preschools allocated with one of the nutrition education strategies verified in this study (DG, SB or SBS), and all the interventions were conducted simultaneously. The intervention was carried out by three groups of researchers and the main researcher was present in all groups. The DG consisted of five mini-games and each of these had a vegetable superhero (tomato, purple cabbage, cucumber, carrot and lettuce) associated with it. Besides, the DG included tailored audio messages about the characteristics and health benefits of these vegetables. The SB was made up of five chapters and each one had a vegetable superhero, equal to the DG, and it was clear in the story the characteristics and functions of the vegetables.

In the group of SBS, the educational sessions were based on the SB and children received a reward (sticker) when they ate the vegetables at the end of the session.

The control group realised educational sessions with the PFWG, the gold standard tool in nutrition education in Portugal, and it was used to promote the group of vegetables. At the end of each session, a play food was distributed to each child and they had to place it in the right group of the PFWG.

Furthermore, in all groups, each week a real vegetable was distributed (tomato, purple cabbage, cucumber, carrot or lettuce), according to the vegetable that was in the DG or the SB, allowing each child to explore sensorially the vegetable.

At the end of each educational session, in all groups, the five vegetables cut in similar portions were offered one after the other to each child. The order to offer the five vegetables was different during the 5 weeks of intervention. Children could serve themselves from a shared plate twice and eat the number of portions they wanted each time. The procedure was consistent across the preschools. Classroom staff were required not to motivate or congratulate children for eating vegetables. The vegetables for the study were all provided and prepared by the research team.

### Intake assessment procedure (baseline, educational sessions, post-test and follow-up)

One week before the intervention began (baseline), children were offered the five vegetables cut or presented in similar portions: one cherry tomato = 1 portion, one stick of cucumber = 1 portion, one stick of carrot = 1 portion, one piece of lettuce = 1 portion and five pieces of grated purple cabbage = 1 portion. Researchers registered the number of portions that each child ate for each assessment. This measurement of vegetable intake was made at baseline (week 1), after each educational session (weeks 2–6), at post-intervention (week 7) and follow-up (week 29). All measurements of vegetable intake were made by researchers, and the time of the intake assessment procedure was agreed upon with the preschool staff. The primary outcome was the vegetable intake. This was verified by the difference in the number of portions consumed from baseline (week 1) to post-intervention (week 7) and from baseline to follow-up (week 29).

### Statistical analysis

All analyses were conducted using the statistical package for the social sciences (SPSS) version 26. Pearsons’ χ^2^ tests and one-way ANOVA were used to analyse socio-demographic differences between the four educational strategies at baseline, post-test and follow-up.

Repeated measures ANOVA was used to detect any univariate differences in vegetable intake, between groups, time (baseline, post-intervention and follow-up) and group by time. For each analysis, the main effect for group, the main effect for time and interaction between group and time are reported in Table 4. Bonferroni contrast analysis was used to detail specific differences. Where significant interactions were detected, the time and group values were plotted according to the outcome variable to illustrate the interaction.

To allow comparisons of effect sizes across different measures and studies, partial η^2^ effect sizes were calculated for differences in time, group and time-group. Partial η^2^ values of 0·01, 0·06 and 0·14 were applied to determine small, moderate and large effects, respectively^(24)^. All analyses were set at 0·05 significant level.

## Results

### Sample characteristics

A total of 180 children were recruited for the study. Of these, 18 caregivers declined participation and a total of 162 children were enrolled in the study (% of caregivers who refused = 10 %). All groups had similar proportions of females and males (*P* = 0·821) and a higher number of 5-year-old children but without statistical significance (*P* = 0·094). The group that had a higher number of children aged 5 years was the DG group. The number of household elements in the four groups did not differ significantly (*P* = 0·261). However, the group of DG had more caregivers with university education (*P* < 0·001) (Table 2).

**Table 2:** Characteristics of preschool-aged children and caregivers allocated to one of the nutrition education strategies

	Digital game (DG)		Storybook (SB)		Storybook and stickers (SBS)		Control group		Total		*P*-value
	*n*	%	*n*	%	*n*	%	*n*	%	*n*	%	
Total	39	24·1	40	24·7	46	28·4	37	22·8	162	100	
Child’s sex											0·821*
Male	20	51·3	18	45·0	25	54·3	20	54·0	83	51·2	
Female	19	48·7	22	55·0	21	45·7	17	46·0	79	48·8	
Child’s age (years)											0·094*
3	2	5·1	6	15·0	14	30·4	4	10·8	26	16·0	
4	9	23·1	8	20·0	11	23·9	12	32·4	40	24·7	
5	20	51·3	17	42·5	15	32·6	12	32·4	64	39·5	
6	8	20·6	9	22·5	6	13·0	9	24·3	32	19·8	
Caregivers educational level											0·001*
Basic education	1	2·9	9	28·1	6	14·0	4	12·9	20	12·4	
High school	4	11·4	16	50·0	14	32·6	17	54·8	51	31·5	
University education	30	85·7	7	21·9	22	51·2	10	14·5	69	42·6	
Household elements											0·261*
2 elements	1	2·9	1	3·6	2	4·9	3	9·7	7	4·3	
3 elements	14	40·0	7	25·0	19	46·3	8	25·8	48	29·6	
4 elements	19	54·3	15	53·6	14	34·1	17	54·8	65	40·1	
5 or more elements	1	2·9	5	17·9	6	14·6	3	9·7	15	9·3	
Child’s age (years)											
Mean	4·87		4·73		4·28		4·70		4·63		0·031†
sd	0·801		0·987		1·047		0·968		0·977		
Caregivers age (years)											
** **Mean	38·43		35·72		35·84		36·94		36·76		0·089†
sd	4·730		4·747		5·332		5·278		5·090		

* Chi-square test.

† *t* test.

### Intervention effects – frequency of vegetables consumption

At baseline, the children who were allocated to the control group ate more portions of lettuce and carrot (*P* < 0·001) than children in the other groups. Besides, children in the control group had significantly higher consumption of purple cabbage and cucumber than children in the DG group and the SB group (*P* = 0·025, *P* = 0·018, respectively), and a higher consumption of tomato compared with the SB group (*P* = 0·047). As previously mentioned, the procedures were consistent across the preschools and the amount of vegetables served was the same at every centre.

At post-test, children who belong to the control group ate more portions of carrot (*P* < 0·05) than children in the other groups. There were no differences found for other vegetables and groups.

At follow-up, children who belong to the SB group ate more portions of carrot than children who belong to the SBS group (*P* = 0·018). Children in the control group also had significantly higher consumption of purple cabbage than children in the SBS and the SB group (*P* = 0·028, *P* = 0·039 respectively), as presented in Table 3.

**Table 3:** Vegetables consumption at baseline, post-test and follow-up in the four groups (DG, SB, SBS and control group). The number of portions is represented as mean and sd

	Number of portions					
	Baseline		Post-test		Follow-up	
	Mean	sd	Mean	sd	Mean	sd
Lettuce						
DG	0·357	0·497	2·286	1·816	1·570	1·785
SBS	0·750	0·444	3·000	3·162	1·500	1·539
SB	0·421	0·507	1·737	1·727	2·740	3·034
Control group	1·438	0·964	4·125	5·097	2·560	2·756
Carrot						
DG	0·786	0·426	4·500	3·858	4·140	4·470
SBS	1·150	1·089	5·250	4·459	3·500	2·259
SB	1·158	0·898	5·263	3·588	7·680	5·132
Control group	3·250	1·653	9·500	5·215	6·130	4·717
Purple cabbage						
DG	0·786	0·699	2·786	3·468	1·070	1·141
SBS	1·000	2·317	3·250	5·025	0·850	1·226
SB	0·526	0·697	4·000	4·655	0·950	1·393
Control group	2·625	3·304	5·125	10·340	4·130	6·541
Cucumber						
DG	0·357	0·633	1·857	2·685	2·790	3·378
SBS	1·350	1·565	5·200	5·550	3·900	3·796
SB	1·053	1·471	3·211	2·637	3·470	3·991
Control group	2·062	1·948	4·813	4·651	5·060	4·234
Tomato						
DG	0·714	0·914	1·786	2·694	2·640	5·733
SBS	0·850	1·182	4·300	6·174	2·700	4·143
SB	0·737	0·806	2·895	3·160	2·420	3·220
Control group	2·062	2·351	3·188	3·799	3·750	5·145

DG, digital game; SB, storybook; SBS, storybook and stickers.

**Table 4:** Regression model of vegetables consumption at baseline, post-test and follow-up in the four groups

	Main effects (F-value)			
	Time	Group	T × G	Bonferroni
Lettuce	15·856*** η^2^ = 0·196	2·173* η^2^ = 0·091	1·483 η^2^ = 0·064	1st < 2nd, Fup
Carrot	50·308*** η^2^ = 0·436	4·804** η^2^ = 0·181	4·099** η^2^ = 0·159	1st < 2nd, Fup
Purple cabbage	8·991** η^2^ = 0·122	2·173 η^2^ = 0·091	0·355 η^2^ = 0·016	1st < 2nd, Fup
Cucumber	28·794** η^2^ = 0·307	1·991 η^2^ = 0·084	1·103 η^2^ = 0·048	1st < 2nd, Fup
Tomato	11·293** η^2^ = 0·148	0·585 η^2^ = 0·026	1·047 η^2^ = 0·046	1st < 2nd, Fup

* *P* < 0·1, **P < 0·01, ***P < 0·001.

A significant time-by-group interaction was only observed for carrot consumption, with a large effect size (η^2^ = 0·159), as presented in Table 4. There was no significant time-by-group interaction for lettuce, purple cabbage, cucumber and tomato. However, for all vegetables, there was a significant increase in consumption from baseline to post-intervention and follow-up (*P* < 0·001).

**Fig. 2:**
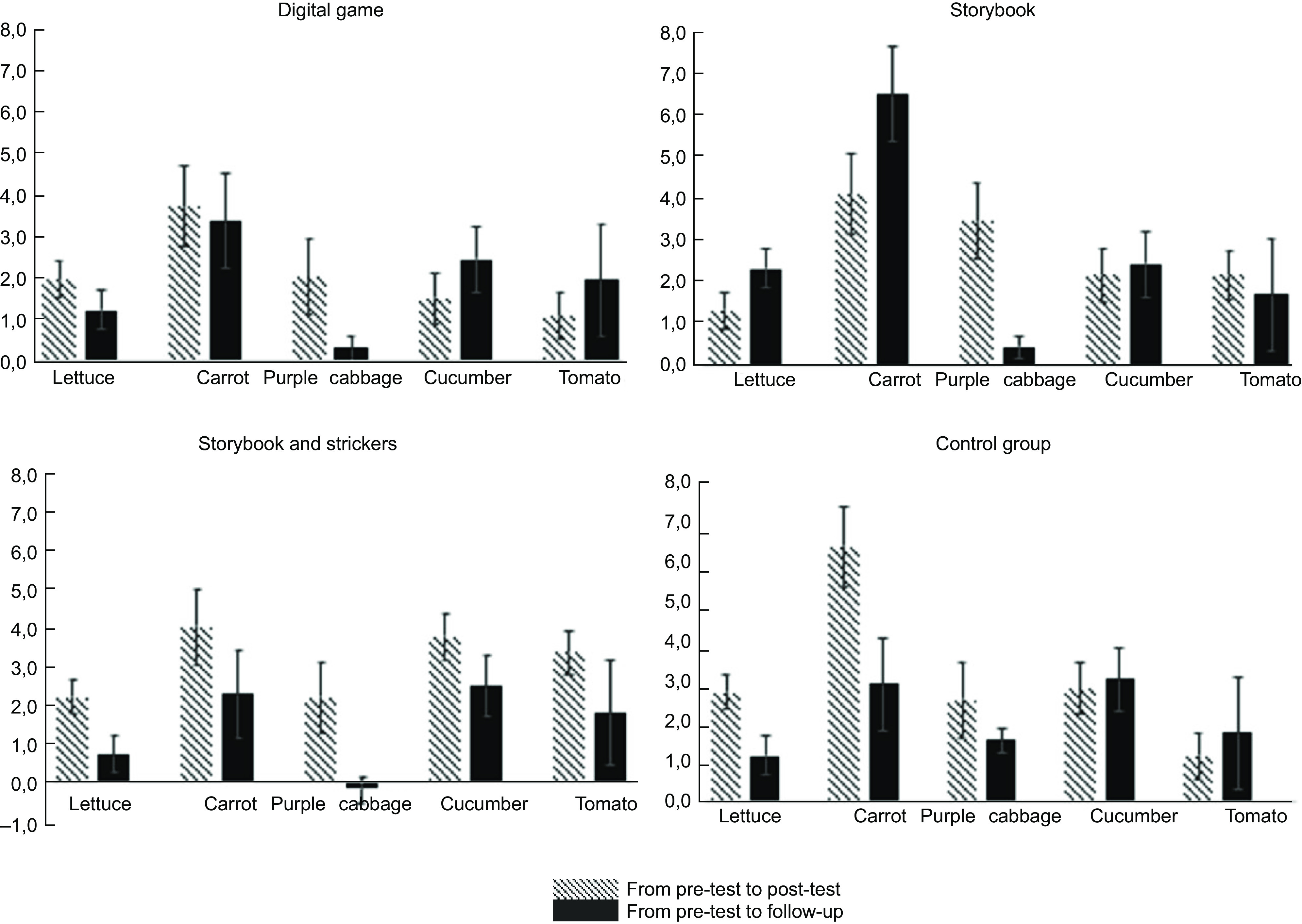
Vegetable consumption variation in the four groups (digital game, storybook, storybook and stickers, and control group) at two different times: from pre-test to post-test and from pre-test to follow-up. From pre-test to post-test; from pre-test to follow-up

In Figure 2, it is possible to observe the variation in vegetable consumption from the moment of the post-test to the baseline (delta 1) and from the follow-up to the baseline (delta 2). In the group of the DG, it was possible to verify a tendency to increase the consumption in the post-test and in the follow-up. For all vegetables, both deltas are positive, which means that there was an increase in consumption. This increase is greater in delta 1 (the difference between post-test and baseline) in purple cabbage but without statistical meaning (*P* = 0·07).

In the SBS group, it was also possible to verify that lettuce, carrot, cucumber and tomato had a tendency to increase the consumption in the post-test and in the follow-up compared to baseline. The greatest variation in consumption since the baseline for the post-test were carrot, cucumber and tomato. The difference in deltas variation in lettuce, purple cabbage and tomato was statistically significant (*P* = 0·048, *P* = 0·040 and *P* = 0·047, respectively). However, it should be noted that delta 2 in purple cabbage consumption was negative, that is, children ate less purple cabbage on the follow-up than on the baseline.

In the SB group, there was a positive variation in the consumption of all vegetables in the two periods analysed. The variation (delta 2) in the consumption of lettuce, carrot and cucumber was higher, and the variation in carrot consumption in the follow-up had a higher statistically significant difference (*P* = 0·032). The purple cabbage also had a statistically significant difference, although there was a decrease in consumption compared to the post-test (*P* = 0·006).

In the control group, there was also a positive variation in the consumption of all vegetables at all times. The carrot was the vegetable that stood out because there was more variation in its consumption, in the follow-up the consumption is not so higher than in the post-test and this variation showed statistical significance (*P* = 0·012).

## Discussion

To our knowledge, this is the first study to analyse an intervention that simultaneously integrates sensory learning (during the intervention, the different vegetables were given to the children for their tactile, sound and visual exploration), repeated taste exposure, reward offer (sticker), the offer of several vegetables simultaneously and learning about the respective vegetables through educational sessions with different strategies (DG and SBS).

Our results show that any of the interventions were effective in increasing vegetable consumption in the short and medium term (post-test and follow-up). The results are consistent with the findings of Nekitsing *et al.* ^(3,18)^, Smith *et al.* ^(22)^, Choi *et al.* ^(25)^ and Sigman-Grant *et al.* ^(26)^ who reported that interventions with educational sessions at the classroom and repeated taste exposure are associated with increases in children’s vegetable intake and more ability to distinguish healthy foods.

At baseline, the control group had a higher consumption of almost all vegetables compared to the other groups, although they did not show significant differences in caregivers characteristics. However, this group was the only one that knew one of the researchers prior to the intervention, because the researcher had a family member in this preschool previously and participated in the assessments of this study, which may refer to the influence of familiarisation with the actors of the food education sessions on their success. To our knowledge, the studies undertaken so far do not assess the influence of stakeholder familiarisation on food education sessions, although it seems an important aspect to be analysed in these types of studies. The intervention developed by a researcher who is unfamiliar to children, by the teacher in a classroom setting or by a familiar relative may have different results that should be understood^(27)^. Regarding the caregivers, we observed more of an absence in returning the informed consent than a deliberate refuse to participate. To the best of our knowledge, this variable is very much influenced by the teacher–caregiver relation and also by the involvement of caregivers in school daily activities. In the schools where teachers were highly proactive, the delivery of informed consent by caregivers was also high. In the schools with less active teachers, the informed consents were handed to the caregivers but never returned.

The carrot was the most consumed vegetable by children in both the post-test and the follow-up, in all groups, although in the baseline (baseline) it was not the most consumed vegetable. There is a consensus that the theory of sensory processing (processing of visual, olfactory and tactile characteristics) is an important contribution to the acceptance of food at an early age^(28)^, which may explain the better acceptance of carrots in our study.

The impact of visual exposure to a certain food through picture books has been widely studied in children at an early age. Tactile exploration of books and the involvement of the child with the actors in the story are crucial factors for the quality of educational programmes for preschool children^(17)^. Previous studies indicate that children’s stories/picture books with vegetables increase the willingness to taste vegetables in children^(3,29–33)^. Nekitsing et al. analysed the effect of using a children’s story with a vegetable that was unfamiliar to the child associated with its sensory play, concluding that there was an increase in the likelihood of children eating this vegetable, but did not reveal any effect on the quantity of the vegetable consumed^(3)^. According to a systematic review by Nekitsing et al.^(20)^, the increase in vegetable consumption is greater when food education sessions are associated with sensory exploration activities of these foods. In our study, the group of children who listened to the story about the five vegetables significantly increased vegetables consumption, reiterating the importance of repeated taste exposure at the same time as educational sessions with SB.

Intervention in the DG group has also proved effective in promoting vegetables consumption in the short and medium term. DG have been increasingly used in health promotion and, although there is increasing scientific literature supporting the potential of these games to influence food intake or physical activity, there are still few studies with an adequate methodology that assess the impact of these games on both dietary behaviour and food knowledge^(11)^. A review study that analysed the nutrition education resources used in schools identified thirty-two curricula for this purpose, of which only fourteen integrate games into their nutrition education resources^(34)^. Most of the systematic reviews and/or meta-analyses carried out within this framework analyse several games for health promotion, but less nutrition education games are found: one of these reviews found only four nutrition education games^(35)^, another review that evaluated digital interventions only included one nutrition education game^(36)^ and another review included six nutrition education games^(37)^. However, all of these reviews analysed games designed for pre-adolescents, adolescents and/or young adults, which reveals the need to create and evaluate the effectiveness of games used in preschool nutrition education.

Other games designed to promote knowledge/healthy eating habits in children have shown as yet inconsistent results. The DG ‘Feed the alien’, which was played in Dutch schools with children between 10 and 13 years of age and aimed at increasing nutrition knowledge, has shown potential to increase nutrition knowledge in the short term but has not proved to be effective in increasing knowledge or eating behaviour in the follow-up (2 weeks after the intervention)^(38)^. A pilot study conducted in secondary schools in Germany revealed that the serious game ‘Fit, food, fun’ increased the knowledge on nutrition in children and adolescents in the short term, although there was more increase in the group that had a traditional classroom teaching format. However, this study did not assess whether there were changes in dietary behaviour^(39)^. Although there is already some diversity of food-related games, the research carried out so far does not make it possible to clarify which elements of the game or procedures performed in these studies will be most effective in increasing knowledge or changing eating behaviour.

The use of stickers is a type of instrumental feeding that has proven to be effective in overcome initial neophobic behaviour, typical of preschool children in a presence of a new vegetable^(40)^. In our study, purple cabbage was the vegetable less recognised by children during the intervention, which may be related to its lower consumption in all groups. In the SBS group, the use of stickers was advantageous in the short term, but in the follow-up there was a decrease in consumption compared to the baseline, which reiterates the hypothesis that stickers are useful in a first phase but are not sufficient to maintain consumption in the long term.

This study demonstrates that any of the educational strategies associated with repeated exposure is effective to promote vegetable consumption in preschool children. The materials available in the preschools and the motivation of the teachers to use the different materials are important issues to recognise in advance, being determinants to the success of the nutrition education sessions. It will be important to understand in future studies if only repeated exposure, without the use of educational materials, will have the same impact on vegetable consumption. Although we can consider that the main determinant of increased vegetable consumption is repeated exposure, perceiving that no differences were observed between the intervention and control groups, it seems important to reflect on the importance of educational strategies as tools capable of promoting knowledge about healthy eating from the earliest ages. The Knowledge-Attitude-Behavior model, one of the models frequently used in planning community nutrition interventions, is based on the premise that a gain in knowledge leads to changes in attitude, which will result in better eating behaviours^(41)^. In this sense, we believe that the educational strategies used in this study may have a positive impact on the future food choices of these children. In addition, according to the Social Cognitive Theory of Albert Bandura, some knowledge is a possible precondition of effective behaviour change^(42)^. Moreover, WHO recommends that food education and understanding should be incorporated into the curriculum in childcare settings^(43)^.

The limitations of the study are related to the number of children that decreased significantly at follow-up, due to the change of academic year and consequent change of school of some children. Another limitation is related to the fact that each school decided the time of vegetable consumption, according to their dynamics, which could affect children appetite. Furthermore, the effect of schools and the intervention group cannot be differentiated. Another limitation was the number of preschools that declined to participate in the study. An explanation for this may be that schools were contacted in March, at the middle of the school year and several preschools had already calendarised the activities, with no opportunity to include new proposals despite the interest in the project.

More studies are needed to comprehend the effect of these strategies in vegetable consumption at home and to test the importance of the interveners in the success of nutrition education sessions.
